# Cellular Output and Physicochemical Properties of the Membrane-Derived Vesicles Depend on Chemical Stimulants

**DOI:** 10.1021/acsami.4c07234

**Published:** 2024-09-09

**Authors:** Dilip Shrestha, Yusuf Bahasoan, Christian Eggeling

**Affiliations:** † MRC Human Immunology Unit, Weatherall Institute of Molecular Medicine, University of Oxford, Oxford OX3 9DS, U.K.; ‡ Department of Life Sciences, Imperial College London, London SW7 2AZ, U.K.; § Department of Biophysical Imaging, Leibniz Institute of Photonic Technologies e.V., member of the Leibniz Centre for Photonics in Infection Research (LPI), Albert- Einstein Strasse 9, 07745 Jena, Germany; ∥ Institute of Applied Optics and Biophysics, Friedrich Schiller University Jena, Max-Wien Platz 1, 07743 Jena, Germany; ⊥ Jena Center for Soft Matter (JCSM), Philosophenweg 7, 07743 Jena, Germany

**Keywords:** plasma membrane vesicles (PMVs), DTT, NEM, lipid order, fluorescence correlation spectroscopy (FCS)

## Abstract

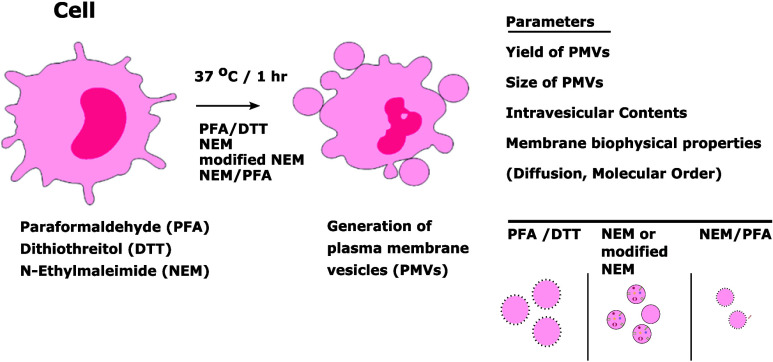

Synthetic liposomes are widely used as drug delivery vehicles in biomedical treatments, such as for mRNA-based antiviral vaccines like those recently developed against SARS-CoV-2. Extracellular vesicles (EVs), which are naturally produced by cells, have emerged as a next-generation delivery system. However, key questions regarding their origin within cells remain unresolved. In this regard, plasma membrane vesicles (PMVs), which are essentially produced from the cellular plasma membrane (PM), present a promising alternative. Unfortunately, their properties relevant to biomedical applications have not be extensively studied. Therefore, we conducted a thorough investigation of the methods used in the production of PMVs. By leveraging advanced fluorescence techniques in microscopy and flow cytometry, we demonstrated a strong dependence of the physicochemical attributes of PMVs on the chemicals used during their production. Following established protocols employing chemicals such as paraformaldehyde (PFA), *N-*ethylmaleimide (NEM) or dl-dithiothreitol (DTT) and by developing a modified NEM-based method that involved a hypotonic shock step, we generated PMVs from THP-1 CD1d cells. We systematically compared key parameters such as vesicle output, their size distribution, vesicular content analysis, vesicular membrane lipid organization and the mobility of a transmembrane protein. Our results revealed distinct trends: PMVs isolated using NEM-based protocols closely resembled natural vesicles, whereas PFA induced significant molecular cross-linking, leading to notable changes in the biophysical properties of the vesicles. Furthermore, our novel NEM protocol enhanced the efficiency of PMV production. In conclusion, our study highlights the unique characteristics of chemically produced PMVs and offers insights into their potentially diverse yet valuable biological functions.

## Introduction

Lipid-based synthetic nanoparticles, also known as liposomes, are widely used as drug carriers against multiple diseases,^1^ such as cancer and microbes. They were an integral part of vaccine formulations in the fight against coronavirus disease 2019 (COVID-19), which were highly successful in preventing deaths related to this disease.^2,3^ Among the main advantages of liposomes are their simple design, tunable composition, and the ability to be produced in a controlled manner.^4^ However, bioavailability and efficient delivery of cargo to the targeted tissues and organs of interest remain a problem with these carrier systems.^4^ Their performance has been considered unsatisfactory due to passive targeting leading to nonspecific uptake by cells, resulting in side effects from unwanted immune responses.^5,6^ Natural vesicles produced by all cells, termed extracellular vesicles (EVs),^6^ have emerged as a promising alternative to liposomes due to their membrane molecular composition and advantages related to their cellular origin. For example, EVs are known to be biocompatible with favorable pharmacokinetics. They inherit cellular lipids and integral membrane receptors that are believed to provide them with intrinsic tissue-homing capacity, allowing them to efficiently interact with the tissues and organs.^4,6^ Despite considerable interest, EV research has been plagued by technological shortcomings and gaps in knowledge regarding their cellular origin. Specifically, EVs are known to be a highly heterogeneous population with varied cellular properties. A promising remedy for these issues is plasma membrane vesicles (PMVs), which are exclusively produced from the cellular plasma membrane (PM). Their membrane composition closely resembles the host cell’s PM, resulting in a fairly homogeneous population. Giant plasma membrane vesicles (GPMVs), which are micron-sized PMVs, have been instrumental in studying the biophysics of the PM, playing a crucial role in establishing the relevance of membrane heterogeneity and the functional role of lipids in the organization of PM.^7−11^ These studies revealed that GPMVs closely mimic the molecular compositional complexity of the PM. They harbor all essential features found in EVs, and most importantly, their source of origin is well-defined. Yet, limited studies have explored the possibility of using them in biomedical applications^12,13^ or as tools for drug delivery or in immunotherapy.^14,15^ Hereon, we refer to any-sized vesicles, including GPMVs, which are generated during the production process, as PMVs.

Production methods for PMVs are well-established but not fully characterized for their biomedical use.^10,16,17^ Most importantly, there is limited information regarding the large-scale production of pure PMVs.^18,19^ PMVs are produced using chemicals such as paraformaldehyde (PFA), dithiothreitol (DTT), *N*-ethylmaleimide (NEM) or ethanol.^7,10,16^ These are highly reactive chemicals that may alter the functions of molecules and the overall biophysics of the PM. Their mechanisms of action are very specific: PFA is a cross-linking reagent used as a fixative for the immobilization of proteins, functioning mostly by modifying their amino and sulfhydryl groups;^20^ DTT is widely used in biochemistry as a reducing agent for breaking disulfide bonds; NEM modifies thiol groups;^21,22^ and ethanol is a polar solvent that alters membrane properties.^23,24^ PMVs produced using these chemicals might therefore differ in their membrane biophysical and physicochemical properties. This became evident in studies highlighting differences in the biophysical behavior of lipid phases^8,25^ and the partitioning of the LAT (linker for activation of T cells) protein^10^ in PMVs isolated from cells using PFA/DTT and NEM. Despite these differences, mobility of lipids in the membrane of these PMVs were found to be similar.^11^ However, aggregates of proteins in the membrane of PMVs were noted in the NEM preparation.^26^ In fact, lipid tubulation networks have been reported in PMVs generated using NEM but not in the vesicles formed from PFA/DTT.^27^ These studies clearly show that chemicals can affect the membrane properties of PMVs.^8^ Yet, a detailed investigation comparing physicochemical and biophysical properties of PMVs based on isolation procedures and their effects on yield and suitability for subsequent biological applications is lacking. Additionally, previous experiments were mainly performed using microscopy, which required only a few isolated manually selected PMVs for analysis.

In this study, we performed a series of experiments utilizing a wide range of techniques including flow cytometry, confocal microscopy and fluorescence spectroscopic techniques to elucidate the differences in the physicochemical and biophysical properties of PMVs generated by the different methods. We found flow cytometry to be an efficient method for characterizing yield and production efficiency, size distribution, and intravesicular contents of the PMVs. Further, we employed the environment-sensitive fluorescent membrane probe, C-laurdan, in generalized polarization (GP) fluorescence measurements. The GP value quantifies shifts in the fluorescence emission spectrum of C-laurdan to assess molecular packing or ordering of its lipid membrane environment, disclosing potential phase-separation into loosely packed liquid-disordered (Ld) and more tightly packed liquid-ordered (Lo) regions.^28,29^ GP measurements thus allow robust quantification of any alterations in the physicochemical and biophysical features of the PMV membrane due to changes in lipid composition or hydration level and differences in molecular order. Finally, amino residues and thiols (sulfhydryl-groups) are, as already highlighted, the primary targets for PMV-inducing chemicals which are abundantly found in proteins. Therefore, we sought to determine the influence of these chemicals on the mobility of a membrane protein in PMVs, employing fluorescence correlation spectroscopy (FCS).^30^ Specifically, we measured the mobility of a PM-embedded receptor, the Cluster of differentiation 1d (CD1d) protein, which has been shown to play a crucial role in the development of immune responses against pathogens.^31^ As expected, we found distinct differences in the properties of the PMVs based on isolation methods and chemicals. Our GP measurements and FCS results were complementary and supported the observed effects. Subsequently, we also developed a novel method to more efficiently produce PMVs using NEM and a hypotonic shock step and compared it with the traditional methods. In conclusion, our study suggests that all PMVs are not alike, and their physicochemical and biophysical properties depend on the chemical methods used in their production. Furthermore, PMVs produced through various preparative methods might offer distinct advantages over synthetic systems. However, the biophysical properties of the PMVs produced via the NEM-based methods make them a superior alternative choice for biomedical purposes.

## Materials and Methods

### Cell Lines

We used THP-1 CD1d cells, which have been fully characterized and previously used in immunological studies.^32^ These cells were kindly provided by Dr. Mariolina Salio from the MRC Weatherall Institute of Molecular Medicine, University of Oxford, U.K. This cell line was virally transduced for stable expression of an immunologically relevant transmembrane receptor, CD1d.^33^ THP-1 CD1d cells were maintained at a density of 0.6–0.8 × 10^6^ cells/mL in a 25 mL flask at 37 °C in an incubator supplied with 5% CO_2_. Complete growth media was used for culturing these cells, prepared by supplementing RPMI 1640 basal media (Sigma-Aldrich, U.K.) with 2 mM l-glutamine (Sigma-Aldrich, U.K.), 1% penicillin-streptomycin (Sigma-Aldrich, U.K.) and fetal calf serum [10% (v/v), Sigma-Aldrich, U.K.].

### Chemicals

The following chemicals were used in this study: NEM, DTT, PFA (16%, methanol-free Electron microscopy grade), Acridine orange (AO), HCS NuclearMask Red Stain (NMRS) and Calcein AM (hereafter Calcein) were all purchased from Thermo Fisher Scientific, U.K.; *N*-2-hydroxyethylpiperazine-*N*′-2-ethanesulfonic acid (HEPES), calcium chloride (CaCl_2_) and sodium chloride (NaCl) were obtained from Sigma-Aldrich, U.K.; and the membrane probe C-laurdan was acquired from 2pprobes (Seoul, South Korea).

### Buffers

Hypotonic and isotonic buffers of pH 7.4 were prepared using HEPES, CaCl_2_, and NaCl. The final concentrations of HEPES and CaCl_2_ were 10 mM and 2 mM respectively. For the hypotonic buffer, 50 mM NaCl was used, whereas 150 mM NaCl was used for the isotonic buffer.

### Vesiculation of Cells

Vesicles were prepared from THP-1 CD1d cells following protocols described in earlier publication.^16^ Instead of keeping the cells in an incubator, we generated PMVs in a thermomixer maintained at 37 °C at a constant speed of 450 rpm for 60 min. Isotonic buffer was used in the PFA and DDT treatment (PFA/DTT) method, whereas hypotonic buffer was used in the conventional NEM method. In our experience, the vesiculation efficiency of the conventional NEM method was not as good as that of the PFA/DTT method, especially in the case of suspension cells such as Jurkat T cells. Therefore, we also modified these protocols and developed new methods to compare their physicochemical features. We introduced a hypotonic shock step in the modified NEM method. In this case, cells were prepared for vesiculation in the following manner: isotonic buffer wash, hypotonic buffer wash, incubation in the hypotonic buffer for 2 min, and finally suspension of cells in the isotonic buffer for vesiculation. The effect of PFA in the modified NEM method was also examined. For this purpose, we added 25 mM PFA into the 2 mM NEM-containing vesiculation buffer, i.e., NEM/PFA method.

### Labeling of Cells and Vesicles with AO or Calcein and/or NMRS Dyes

AO and Calcein are membrane-permeable dyes.^34^ PMVs and cells can be labeled before or after vesiculation. However, we preferred labeling them after vesiculation to avoid any secondary effects of dyes on the vesiculation process. AO and Calcein were added to the solution containing cells and PMVs at a final concentration of 50 μM and 10 μM respectively. For AO, ∼5 min of incubation at room temperature (RT) was sufficient, whereas Calcein was incubated for 20 min at 37 °C. Cells were labeled with the NMRS dyes before vesiculation by incubating them with the dye (1:500 dilution) for 20 min at RT. They were washed twice with the isotonic buffer before proceeding with the vesiculation steps.

### Labeling with C-Laurdan

Labeling of PMVs with C-laurdan was performed by adding ∼0.5 μM of the probe to the PMV solution and incubating for ∼2 min at RT. The vesicles were then transferred to Ibidi glass chambers (#1.5) suitable for high-resolution microscopy.

### Flow Cytometry for Analysis of Cells and Vesicles

For quantitative analysis of PMVs, Attune^TM^ NxT flow cytometry from ThermoFisher Scientific was used in this study (flow cytometry facility of the Weatherall Institute of Molecular Medicine, University of Oxford, U.K.). Forward scatter (FSC) and side scatter (SSC) thresholds were set to the minimum that allowed distinct visualization and separation of small vesicles from the scattering noise of the buffer. Acquisition of the sample was set to a speed of 25 μL/s. Calcein and AO were excited with the 488 nm laser, and the fluorescence emitted was collected at the BL1 (530/30) and BL3 (695/40) detectors. Excitation for the NMRS stain was done with a 633 nm laser, and the emission was collected at RL1 (670/14) detector. Attune^TM^ NxT flow cytometry is suitable for volumetric counting measurement; therefore, we fixed the acquisition volume to 50 μL which gave absolute numbers for vesicles or cells in the sample.^35,36^ The flow cytometer settings remained the same throughout the study.

### Confocal Spectral Microscopy

GP measurements were performed with an oil-immersion Plan-Apo 63x/1.4 NA objective on a Zeiss LSM 880 confocal microscope. C-laurdan was excited with a 405 nm laser, and the resulting fluorescence was passed through an optical diffraction grating before being collected using a 32-channel GaAsP detector at a resolution of 8.9 nm from 415 to 695 nm wavelengths. 512 × 512 sized 16-bit files were saved in.lsm format for further analysis.

### GP Analysis

A previously published Fiji/ImageJ compatible GP plugin was used for processing spectral images. The software provides the GP values pixel-by-pixel in an image.^28^ The spectrum obtained from each pixel of an image was Gaussian-fitted, and values were extracted at specific wavelengths for calculating GP based on eq 1.
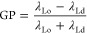
1

We arbitrarily selected the wavelengths 495 (λ_Ld_) and 440 (λ_Lo_) nm corresponding to Ld and Lo regions according to their sensitivity to the respective membrane phases.^28^

### Fluorescence Correlation Spectroscopy (FCS)

FCS experiments were performed on a Zeiss LSM 880 inverted confocal microscope using a 40X C-Apochromat NA 1.2 W Corr FCS objective (Zeiss). Once PMVs were produced from THP-1 CD1d cells, they were briefly centrifuged at 100*g* for 2 min. The lower 200 μL of the sample in the microfuge tube was pipetted out for labeling. Preparation of anti-CD1d Fab antibodies (51.1.3,^37^), and their conjugation to Alexa Fluor 488 dyes was done in the laboratory following standard protocols. These conjugates were then added to the PMV solution, final concentration of 10 μg/mL and were kept at RT for approximately 1 h. Labeled PMVs were then carefully placed on μ-Slide 8-well ibidi chambers with a glass bottom for FCS experiments. Alexa Fluor 488 dyes were excited with a 488 nm laser and the emission signals were collected at wavelengths ranging from 520 to 590 nm. The excitation laser power used was ∼2.1 μW before the objective. Experiments were performed at 25 °C and data were collected for 10 s. The size of the observation spot for FCS measurement was calibrated using Alexa Fluor 488 dyes in water. In consideration of the reported diffusion coefficient (*D* = 414 μm^2^/s) of this dye,^38^ our setup resulted in a full-width at half-maximum (FWHM) of the observation spot of 240 nm and 0.126 ± 0.008 μm^2^ observation area in the lateral dimension. Measurements were performed on the top membrane of PMVs. FCS autocorrelation curves were then fitted with our freely available FoCuS-point software with a one component, two-dimensional (2D) free diffusion model with a triplet component.^39^ The triplet transit time was determined experimentally and was fixed to 3 μs for Alexa Fluor 488 dye. The samples were measured independently on two separate days and the data from at least 15 PMVs were pooled to get the value of the average transit time through the observation spot as a measure of mobility.

The following equation was used for calculating diffusion coefficient (*D*)
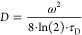
2where “ω” is the FWHM of the observation spot and “τ_D_” is the mean transit time reflecting the time spent by the molecule in the observation spot.

### Statistical Analysis

All analysis was done using GraphPad V9. One way ANOVA was performed followed by the Kruskal–Wallis test to compute statistical differences in the samples. Median, mean and standard deviation (SD) values were also calculated from these data. *P*-values were set at **** (0.0001), *** (0.0002), ** (0.0021) and * (0.0332) respectively.

## Results

We generated PMVs from THP-1 CD1d cells using different protocols: (i) no treatment as a control (natural vesicles), (ii) PFA/DTT treatment, (iii) conventional (pure) NEM treatment, (iv) NEM method involving a hypotonic shock as a novel protocol (modified NEM, see the Materials and Methods section), and (v) NEM/PFA treatment. We determined their cellular output and various physicochemical properties employing different techniques: (1) flow cytometry for PMV yield, their size and intravesicular content, (2) confocal fluorescence microscopy in combination with GP imaging for membrane lipid order, and (3) FCS for CD1d mobility.

### Optimizing Flow Cytometer to Quantify and Characterize PMVs

**Figure 1:**
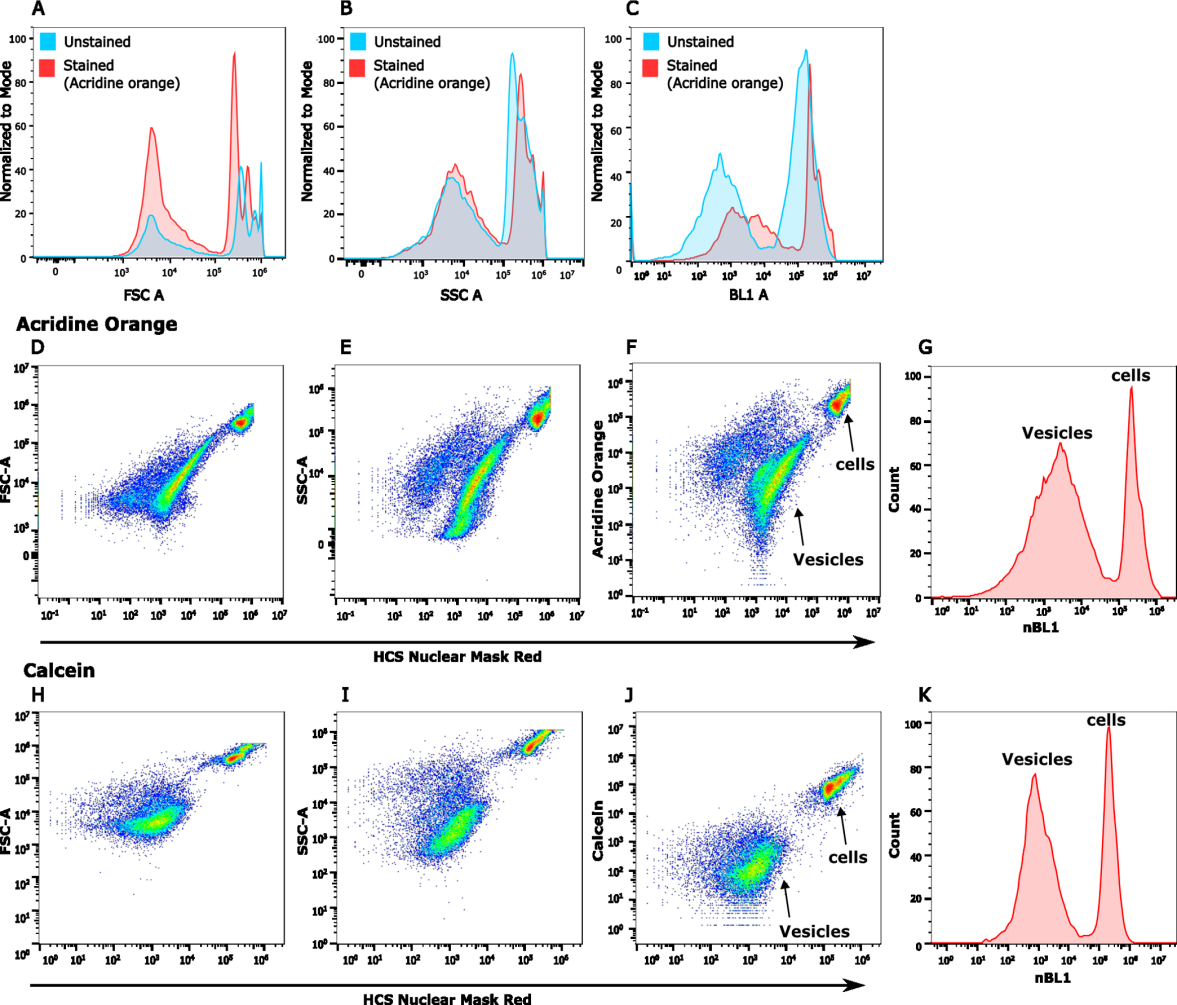
Visualization of membrane vesicles with fluorescent dyes. The first row shows histograms of unstained (faint blue) and AO-stained (red) samples, representing various features of the membrane vesicles and cells: (A) FSC A (Area)—relates to the size of events, (B) SSC A (Area)—relates to the cellular (or vesicular) contents, (C) BL1 A (Area)—fluorescence from AO. The second and third rows show samples stained with AO (or Calcein) and HCS Nuclear Mask Red (or NMRS dye). Scatter plots of FSC A, SSC A and AO (or Calcein) against NMRS dye: AO; panels (D–F) and Calcein; panels (H–J). Histograms of nBL1 (or new BL1), calculated after subtraction of background fluorescence from vesicles for accurate identification of PMVs, are shown in panels (G, K). The vesicle quantification strategy is described in Figure S2; however, it can be easily identified visually in the scatter plot as shown in (F, J), marked with arrows, prior to background subtraction as well.

**Figure 2:**
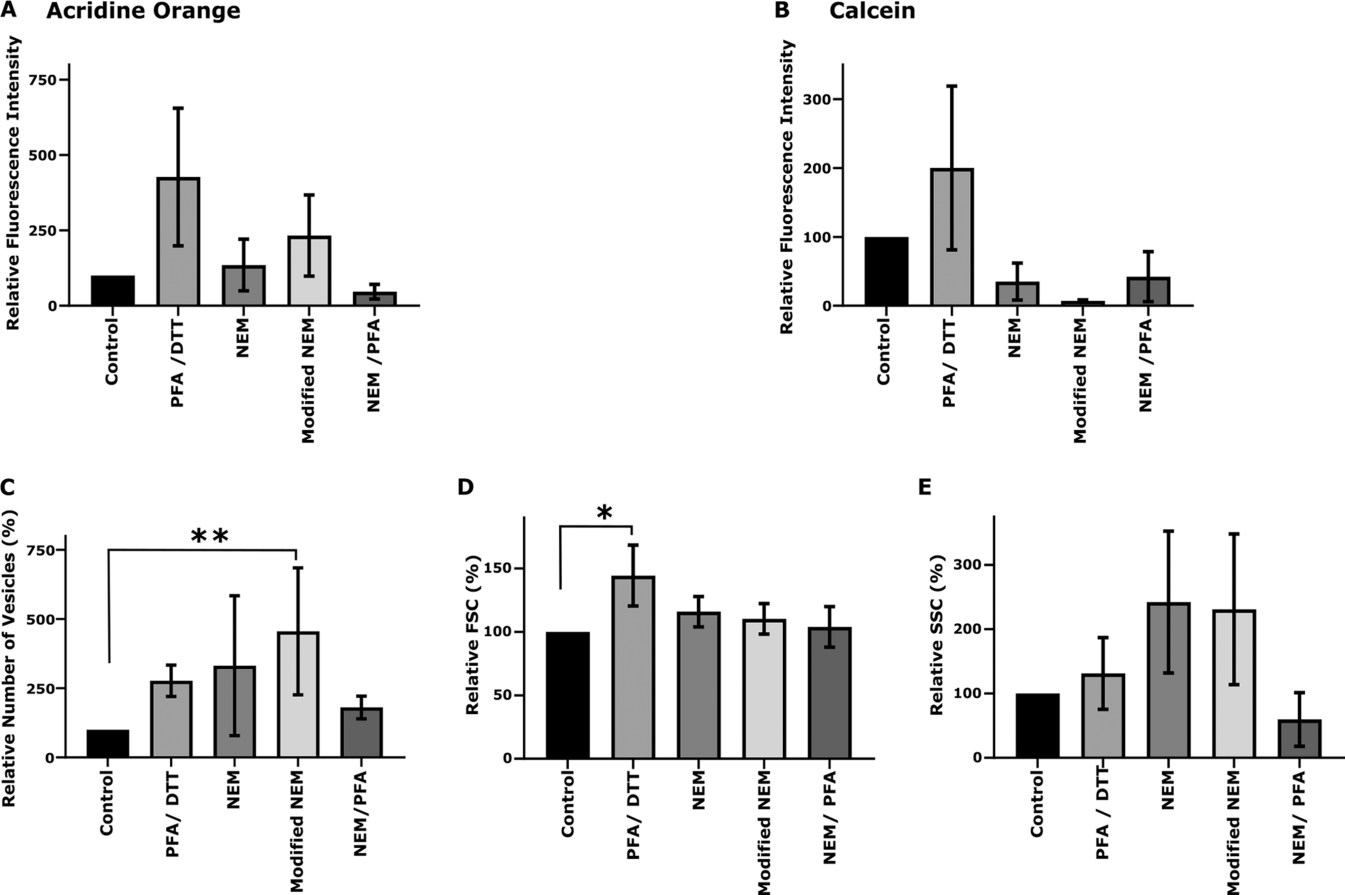
Characterization of fluorescent PMVs. Relative fluorescent intensities of vesicles due to AO and Calcein dyes are shown in (A, B). In this study, AO-stained samples were primarily utilized for quantifying various attributes of vesicles. The number of quantified vesicles, as well as the FSC and SSC features for PMVs produced using various chemical conditions, relative to vesicles from control samples (i.e., natural vesicles produced without chemicals in PMV buffer) are shown in Panels (C–E). At least three independent measurements were performed, and normalization was performed assuming vesicle parameters from control samples as 100% in all cases. Figures are shown as Mean ± SD. For determining level of significance among samples, first one-way ANOVA was performed then multiple comparison tests were done using the Kruskal–Wallis test. Only groups reaching the significance level, *p*-value <0.05, are displayed in the figure. Here, ** and * indicate *p*-values 0.0021 and 0.0332 respectively.

Quantification and size characterization of various objects are often performed using dynamic light scattering (DLS). However, the sizes of PMVs range from submicrons to several microns in diameter, making them too large to be accurately quantified by DLS. In contrast, flow cytometry can straightforwardly analyze objects as large as cells and PMVs. Flow cytometry is a high-throughput technology that can report the population heterogeneity and the physicochemical features of single particles, such as PMVs or cells, on a particle-by-particle basis. Thousands to millions of particles can be rapidly investigated, generating large data sets for statistical analysis. Flow cytometry is used for counting particles, estimating their size and granular content, characterizing their fluorescence labeling efficiency,^40^ quantifying their biophysical properties, such as membrane lipid order in the case of vesicular objects,^41^ and for investigating molecular interactions.^42^ Recently, its use has become popular in the field of EVs which are typically considered smaller than 200 nm in diameter.^43^ Given these capabilities, we employed the Attune^TM^ NxT flow cytometer, which enables straightforward quantification of particles^35^ without requiring reference beads.^44,45^ With the optimized instrumental settings for FSC and SSC, the size resolvability of our system was ∼600 nm, suitable for comparing PMVs (Figure S1). We also investigated whether fluorescently labeling the PMVs facilitated a more sensitive detection. Figure 1A–C depict representative flow cytometry histograms of PMVs with and without fluorescence staining by AO, clearly highlighting the advantage of employing fluorescently labeled PMVs for their characterization in flow cytometry (Figure 1C). Often, lipophilic dyes such as DiO, DiI, PKH67, or PKH26 are used for labeling the membranes of EVs.^46−50^ To avoid biased detection of membrane debris generated during the vesiculation process of PMVs from cells, we chose dyes that would specifically stain the vesicular lumen content. We selected the dyes Calcein and AO, which have previously been used in EV studies.^34,50,51^ Calcein is nonfluorescent unless it is hydrolyzed by an esterase, converting it into a negatively charged entity facilitating its retention in the cytosol and further inside the PMVs. AO is a fluorescent dye specific for nucleic acids. Binding of AO to DNA and RNA gives rise to maximum fluorescence signals at different spectral wavelengths after excitation at 488 nm: green fluorescence around 520 nm for DNA and red fluorescence around 635 nm for RNA.^52^ Additionally, we included the fluorescent nuclear stain NMRS, which greatly enhanced the ability to distinguish PMVs from residual cells. Thus, our general strategy was to use a combination of these nuclear and cytosolic dyes for effective discrimination of PMVs: (i) Residual cells were highly fluorescent from the nuclear stain NMRS, at least 4-fold higher in fluorescence than that of PMVs, and could thus be straightforwardly identified (Figures 1D–K, and S2) and excluded from analysis; (ii) Calcein or AO fluorescence of the remaining events was taken to identify true PMVs. For this, we subtracted the background signal (considered as twice the median value of the corresponding signal from an unstained sample) from the fluorescence levels of Calcein or AO and considered events that still exhibited positive fluorescence signal as vesicles, as indicated by nBL1 in Figure 1G,K. The details on identifying the vesicles are described in Figure S2. Using this strategy, we found that both Calcein and AO were suitable for identifying PMVs, allowing for the distinct visualization of stained PMVs (Figure 1D–K). However, AO was superior to Calcein and resulted in highly fluorescent vesicles, presumably due to the higher quantum efficiency which was shown to increase further upon binding to nucleotides.^53^Figure 2A,B show the relative average fluorescence signal levels determined for Calcein and AO for the PMVs from different treatments. For Calcein, the relative fluorescence intensity in PMVs produced from treatments with conventional NEM, modified NEM and NEM/PFA was lower compared to that of the control sample. In comparison, AO stained well for all treatments except for the NEM/PFA method, where we observed a lower fluorescence intensity than that from the control PMV samples. Following these observations, we decided to employ the combination of AO and NMRS for further studies.

### PMV-Inducing Chemicals Give Rise to Vesicles of Diverse Features

Following the optimization of flow cytometry settings for studying PMVs, we next sought to compare the different features of the produced PMVs.

PMV yield and production efficiency: For the determination of the PMV yield, we normalized the number of vesicles to the number of cells detected during the flow cytometric measurement. The relative yield was thereafter calculated, taking PMVs from the control sample as 100%, as highlighted in Figure 2C. Though distinct differences due to chemicals were noted, a large variation in day-to-day experiments masked these changes. Nonetheless, we could identify a definitive trend: (a) The PMV yield for all the chemical methods was higher than that from the control sample, (b) The highest yield was for our novel optimized modified NEM treatment protocol, followed by the conventional NEM and PFA/DDT treatments, and NEM/PFA treatment yielded the lowest number of PMVs, (c) We have to note that we observed a large fraction of cell-bound vesicles following the conventional NEM methods (Figure S3, third row), indicating the inability of the conventional NEM method to produce detached isolated vesicles, which was resolved by our modification using the hypotonic shock step.

PMV size: We considered FSC values (Figure 2D) as a measure of the relative size of the PMVs, which is a widely used metric in flow cytometry. There were slight differences in size for the different treatments, with PFA/DDT producing the largest vesicles, while treatments involving NEM generated vesicles of similar sizes, just a little bit larger than the control (or natural) vesicles.

PMV intravesicular contents: For this analysis, we took SSC values (Figure 2E) as an estimate for the scattering objects (such as fragments of cellular cytosolic components), i.e., intravesicular contents. While the SSC values also scale with vesicle sizes, they are more strongly determined by scattering, scaling with object contents.^54^ The NEM samples (whether conventional or modified) showed the highest intravesicular contents, while the addition of PFA to the treatment resulted in a clear reduction in this feature, even below control levels of the natural vesicles. The latter can also be observed in fluorescence microscopy images (Figure S3, rows third, fourth, and fifth).

Our observations clearly indicate differences in the PMVs produced by the different methods. They also highlight an optimized yield of PMVs with reasonable sizes for our novel modified NEM treatment.

### Membrane Biophysical Differences Exist in PMVs from Different Methods

**Figure 3:**
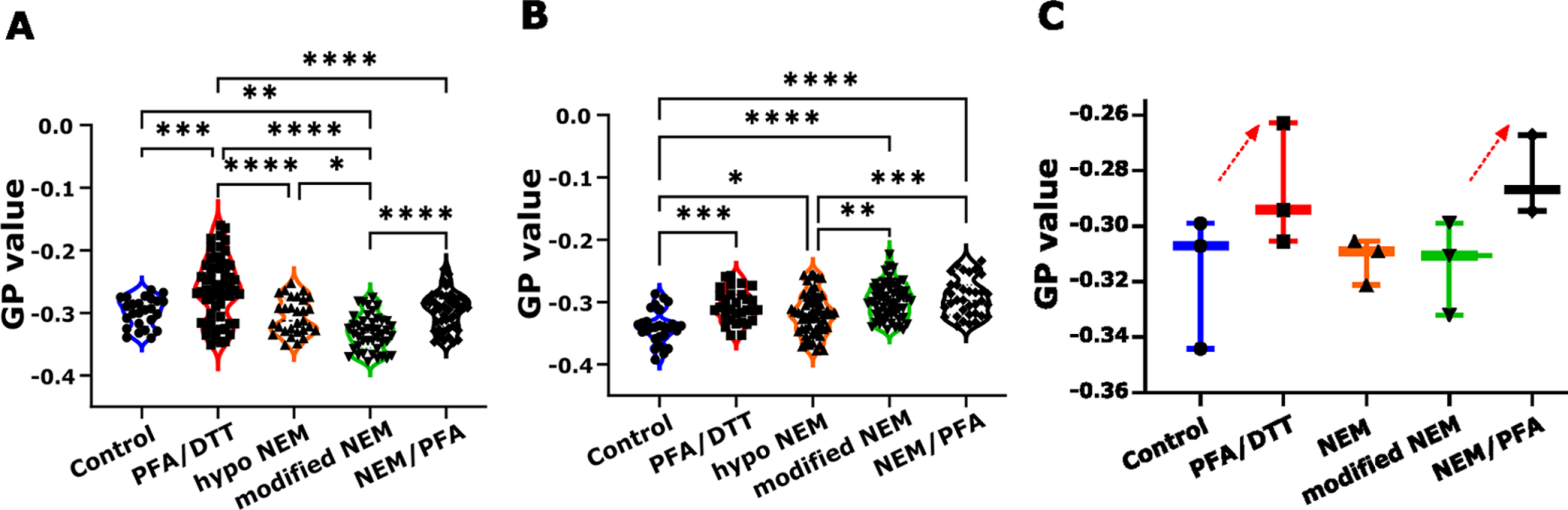
GP measurements using spectral imaging for the PMVs produced by different chemical methods. Panels (A, B) show independent experiments performed on different days, with each dot representing a GP value from a single cell. Statistical analyses were performed using one-way ANOVA, and the Kruskal–Wallis test was employed to determine significant differences among samples for each day. In the figure, *p*-values of 0.0001, 0.0002, 0.0021, and 0.0332 are shown as ****, ***, ** and * respectively. Pairwise comparison statistics between groups that did not meet the significance level, *p*-value <0.05, are not displayed in the figure. The comparison of GP values for PMVs measured on three different days are shown in (C) as a box and whisker plot. Arrows indicate increase in GP value.

Next, we aimed at characterizing the membrane lipid packing of the differently produced PMVs. For this, we employed the membrane probe C-laurdan in combination with confocal spectral imaging and quantified the packing as GP value, as highlighted in the methods. Large GP values indicate dense lipid membrane packing and low GP values indicate less dense and more fluid environments. As with the flow cytometry measurements, the GP values disclosed a significant variability between day-to-day measurements (Figure 3A,B). Still, we noted a trend reflecting an increase in the lipid molecular packing due to PFA treatment (Figure 3C). This is presumably due to its cross-linking activity and follows a feature that is reminiscent of antibody cross-linking activity in membrane molecular packing.^26^ In contrast, it was difficult to distinguish differences in lipid order between PMVs from control (i.e., natural PMVs) and sole NEM (whether conventional or modified) treatments (Figure 3C), although it has to be emphasized that generating vesicles without chemicals are relatively difficult.

### Diffusion of a Membrane Receptor CD1d in PMVs Correlates with GP Measurements

**Figure 4:**
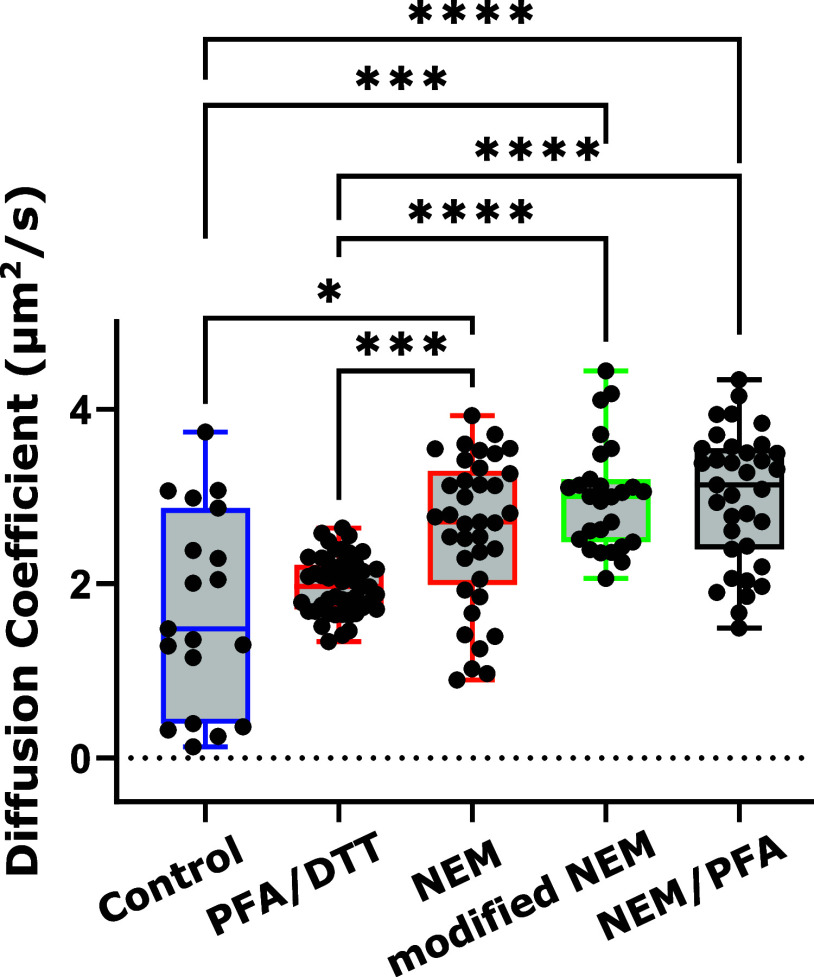
Quantifying diffusion of CD1d receptor on vesicles with FCS. PMVs generated using various chemical methods were stained with Alexa Fluor 488 Fab, which binds to CD1d receptors in the vesicular membrane. Diffusion was measured on the top membrane of these vesicles using FCS. Each dot in the figure represents a curve from a single vesicle. A small number of vesicles were produced under natural conditions, which showed relatively high heterogeneity in diffusion. To compare the data, one-way ANOVA was conducted, and statistical significance was determined using the Kruskal–Wallis test. *P*-values of 0.0001, 0.0002, and 0.0332 are indicated by ****, ***, and * respectively. Nonsignificant differences in pairwise comparison statistics are not shown in the figure.

Given the variations in lipid membrane packing, we next decided to measure differences in the mobility of a membrane protein, specifically the PM receptor CD1d. Well-embedded in the PMV membrane, we labeled CD1d with an organic-dye tagged Fab antibody (avoiding cross-linking of proteins) and performed FCS measurements to determine and compare the diffusion coefficient “*D*” and thus the mobility of CD1d for the differently prepared PMVs (Figure 4). Our results on the mobility revealed high heterogeneity in the diffusion of CD1d, with the highest variability for the natural control vesicles (mean value of *D* = 1.7 ± 1.1 μm^2^/s, i.e., SD of 65%) closely followed by the NEM treatments (SD > 25%). Values of *D* (mean ± SD) for PMVs from NEM, modified NEM and NEM/PFA were 2.7 ± 0.9, 2.9 ± 0.75 and 3 ± 0.76 μm^2^/s, respectively. This variability is not surprising, as CD1d is known to have highly heterogeneous diffusion modes, due to for example strong and heterogeneous aggregation or influences by the membrane-underlying cortical cytoskeleton.^55,56^ Notably, this heterogeneity is significantly reduced following the PFA/DTT treatment (2.0 ± 0.33 μm^2^/s, i.e., SD of 16%), indicating a biased protein mobility. In all cases, chemical treatments led to a slight increase in the mobility compared to the natural vesicles (rise of average *D*-values from 1.7 to slightly above 2 μm^2^/s).

## Discussion

Liposomes have proven to be efficient drug delivery vehicles; however, there is still a growing need for their optimization. Especially alternative candidates to synthetic lipid-based drug delivery platforms are needed that can address inherent disadvantages, such as increased specificity toward diseased tissue. In this study, we have further characterized PMVs, which have already established themselves as an advanced tool suitable for studying various biophysical and physicochemical questions related to the membrane activity.^10,27,57,58^ PMVs are highly favored in such studies due to their similarity to the PM of the source cells.^8−10^ However, only limited studies have so far exploited PMVs in biomedical research or for therapeutic purposes.^14^ To support its suitability for such purposes, we performed a thorough investigation of the well-established chemical methods based on PFA, DTT and NEM and of our novel NEM-based method for generating PMVs. We compared various attributes of the PMVs exploiting advanced fluorescence techniques. Similarities in the conclusions from two cytosolic dyes, Calcein and AO, confirmed the reliability of our method. One can argue about the possibility of vesicle disintegration during the acquisition of data with flow cytometry; however, this would be visible during the data acquisition process as it affects the time-dependent signals in the fluorescence and FSC/SSC channels. The signals from the events were consistent with no detectable changes during the acquisition time window. FSC and SSC settings were chosen to include cells and suppress scattering from the vesiculation buffer, which, however, resulted in a limited resolution of about ∼600 nm when measured with Apogee beads (Figure S1). Resolution of the system can be improved further if vesicles are solely focused, and by optimizing the detector settings, such as the use of more sensitive FSC or SSC detectors. We used thresholds for FSC and SSC to avoid unwanted scattering, but the system can presumably detect smaller particles when the settings are relaxed or ignored,^59,60^ although it may result in bias from buffer-related light scattering. We also demonstrated that dye labeling facilitated more straightforward visualization of vesicles, with both Calcein and AO proving suitable for this purpose. Interestingly, we observed lower fluorescence from Calcein in PMV samples treated with NEM. Since Calcein requires esterase activity to be fluorescent, we presume that NEM inhibits esterase enzymatic activity. However, this assumption requires further investigation. Previous studies have compared the yield of PMVs from PFA/DTT methods by incubating cells for extended periods (>3 h).^18,19^ In this study, we chose to incubate cells for only 1 h, primarily because earlier research showed that cells ceased to produce vesicles beyond this time point.^61^ Additionally, we were concerned about the nature and heterogeneity of vesicles when kept for prolonged incubation with these chemicals. Our approach established that chemicals enhanced the production of PMVs, especially when using our optimized modified NEM approach involving hypotonic shock, and the PFA/DTT protocol produced the largest vesicles.

Based on the SSC channel, our flow cytometry analysis also showed differences in intravesicular components. SSC signals were relatively higher for NEM-based methods, indicating enrichment of contents with high light scattering properties. Surprisingly, PFA suppressed the NEM-induced scattering signals of the PMVs. This suppression might be related to the ability of PFA to fix molecules and thereby preserve intact structures. Our data perfectly align with a recent study that demonstrated the presence of a lipid tubular network in NEM-induced vesicles.^27^ These structures were absent when PFA was added during the vesiculation process, a finding supported by our results with the PFA/DTT or NEM/PFA treatments. Levental et.al.,^8^ revealed that the biophysical properties of the NEM vesicles were distinct from those of the PFA/DTT vesicles since their lipid phase separation properties were different. The characteristics of the vesicles produced by NEM, which revealed high SSC values in our study, suggested that the PMVs produced using these methods might have different lipid compositions. This hypothesis is supported by the results of a recent study on yeast cells, which highlighted lipidome remodeling due to stress on the endoplasmic reticulum within an hour of treatment with DTT.^62^ Fluorescence microscopy using C-laurdan and GP value determination highlighted distinct differences in the membrane biophysical features, namely the membrane lipid ordering, of the differently generated vesicles. The most prominent observation was the effect of PFA treatment in increasing the membrane lipid order. This observation aligns well with the generally known phenomenon that molecular cross-linking or aggregation, e.g., by cholera toxin B or antibodies, results in increased membrane lipid ordering.^26,63^ GP values of PMVs generated from conventional and modified NEM treatments were similar to those of the naturally (nonchemically) produced vesicles, indicating similar membrane biophysical features as previously suggested.^8,27^

Finally, we complemented the GP measurements by quantifying the diffusion of a membrane receptor, CD1d, in the vesicular membrane using FCS. Generally, mobility was highly heterogeneous, as expected for this receptor due to, for example, variable aggregation,^55,56^ except for the PFA/DTT treatment, where we found significantly less heterogeneity in the diffusion characteristics of CD1d. The latter resulted in a characteristic diffusion coefficient with a low SD, presumably because of the uniform cross-linking by PFA and reducing effects of DTT, which might have resulted in a homogeneous distribution of proteins between different phases of the membrane.^8^

Our observations thus suggest that PMVs obtained via different chemicals have different properties. Notably, the NEM treatment, especially when combined with our modified hypotonic shock protocol, produced the highest number of PMVs with biophysical properties similar to those generated naturally, i.e., without chemicals. Regardless of the differences, we believe the molecular complexity exhibited by the PMVs still makes them superior to their synthetic counterparts. For general biological applications, such as gene silencing, PMVs isolated via any of the described methods would potentially serve the desired purpose. However, NEM-based methods might be far more suitable when vesicles representing a specific cellular state are required, as the NEM treatment is less disruptive to cellular processes than the PFA/DTT treatment. NEM treatment is more likely to generate PMVs with membranes reflective of the host cells’ physiological states. For example, NEM-generated PMVs isolated from two distinct functional states of dendritic cells, e.g., immature and mature, would better represent the respective biological characteristics.^64^ Further improvements in the production of PMVs and in methods to quantify them will facilitate their broader biomedical applications, such as the development of therapeutics. A prime example is biomimetic nanoparticles, often used interchangeably with the term nanosponges, which have generated significant interest in biomedical research and therapeutics recently.^65,66^ These particles are formed by coating the polymeric nanoparticles with a PM from a source cell of biological relevance. The development of such hybrid nanosponges from the cellular PM with viral and/or cytokine-binding receptors has been found to interfere with viral infection and reduce associated disorders.^67,68^ To create these particles, cells are homogenized to extract PM. Our proposed methods generate vesicles primarily from the PM, thus providing alternative means for similar purposes. These vesicles could potentially be useful as vaccines^69^ or modified for other therapeutic interventions as well.

## Conclusions

In summary, our study highlighted the unique physicochemical features of PMVs generated using different chemicals. Advantages such as the yield of vesicles and the effects of these chemicals on the biophysical attributes of PMVs, for example, molecular composition or molecular order, revealed their distinct characteristics that would facilitate the adoption of these tools in various biological applications, such as studying mechanisms of virus-host interactions and membrane contacts. Our results also suggested that PMVs generated from the NEM-based methods were very similar to vesicles produced naturally. This method preserves the biophysical properties of the PM, capturing snapshots of cellular physiological states, thereby providing ample opportunities to exploit them in studying biology. In the long run, we believe that with a greater understanding of their biology, PMVs can serve as a next-generation drug delivery system or as an innovative tool that can be solely used for therapeutic benefits.

## Supporting Information Available

Determining size resolution limit of the Attune^TM^ NxT flow cytometry (Figure S1); vesicle identification strategy (Figure S2); fluorescent images of antibody-labeled PMVs (Figure S3) (PDF)
