# Supplementary material for: Cellular Output and Physicochemical Properties of the Membrane-Derived Vesicles Depend on Chemical Stimulants
Source: ACS Appl Mater Interfaces. 2024 Sep 9;16(37):48982–92. doi: 10.1021/acsami.4c07234 (PMC11420866; doi:10.1021/acsami.4c07234)
Supplement: Supplementary file 1 — am4c07234_si_001.pdf [file am4c07234_si_001.pdf]

Supporting Information

Cellular output and Physicochemical Properties of the Membrane-derived Vesicles depend on Chemical Stimulants

Dilip Shrestha[*†], Yusuf Bahasoan, Christian Eggeling[*]

*Dilip Shrestha

MRC Human Immunology Unit, Weatherall Institute of Molecular Medicine, University of Oxford, UK

[†]Department of Life Sciences, Imperial College London, London SW7 2AZ, UK

Email: d.shrestha@imperial.ac.uk

*Christian Eggeling

MRC Human Immunology Unit, Weatherall Institute of Molecular Medicine, University of Oxford, UK

Department of Biophysical Imaging, Leibniz Institute of Photonic Technologies e.V., Albert-Einstein Strasse 9, 07745 Jena, Germany; member of the Leibniz Centre for Photonics in Infection Research (LPI), Jena, Germany

Institute of Applied Optics and Biophysics, Max-Wien Platz 1, 07743 Jena, Germany

Jena Center for Soft Matter (JCSM), Philosophenweg 7, 07743 Jena, Germany

Email: christian.eggeling@uni-jena.de

Author

Yusuf Bahasoan

MRC Human Immunology Unit, Weatherall Institute of Molecular Medicine, University of Oxford, UK

Figure S1

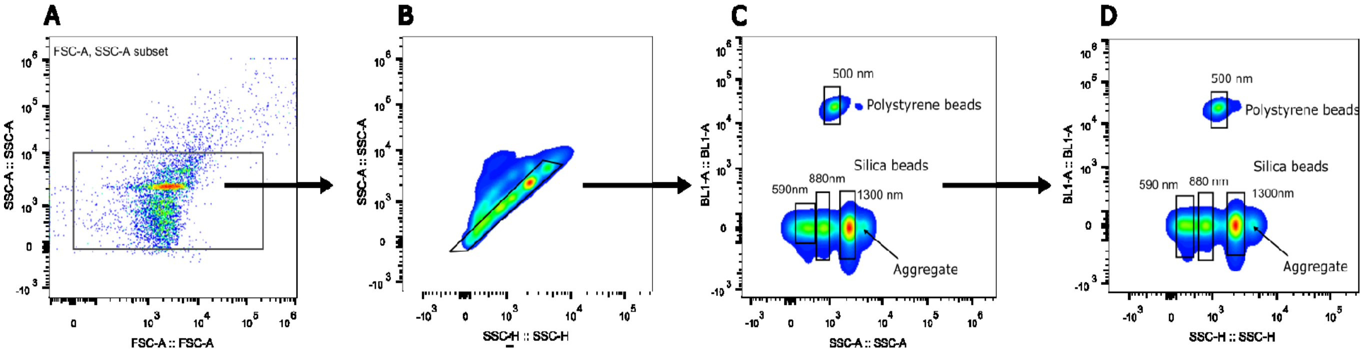

Figure S1. Determining size resolution limit of the Attune^{TM} NxT flow cytometry

Apogee Mix beads were used to determine the resolution limit of the system. The mixture contains non-fluorescent spherical silica beads (180 nm, 240 nm, 300 nm, 590 nm, 880 nm and 1300 nm) and fluorescent spherical polystyrene beads (110 nm and 500 nm). The silica beads have a refractive index of 1.43, which is closer to that of EVs. Panels A, B, C and D show the steps for determining FSC and SSC values. Scatter plots of FSC, SSC or BL1 (green fluorescence) for their signal height (H) and area (A) are shown in these panels. The lower limit for the detection of particles is approximately 590 nm.

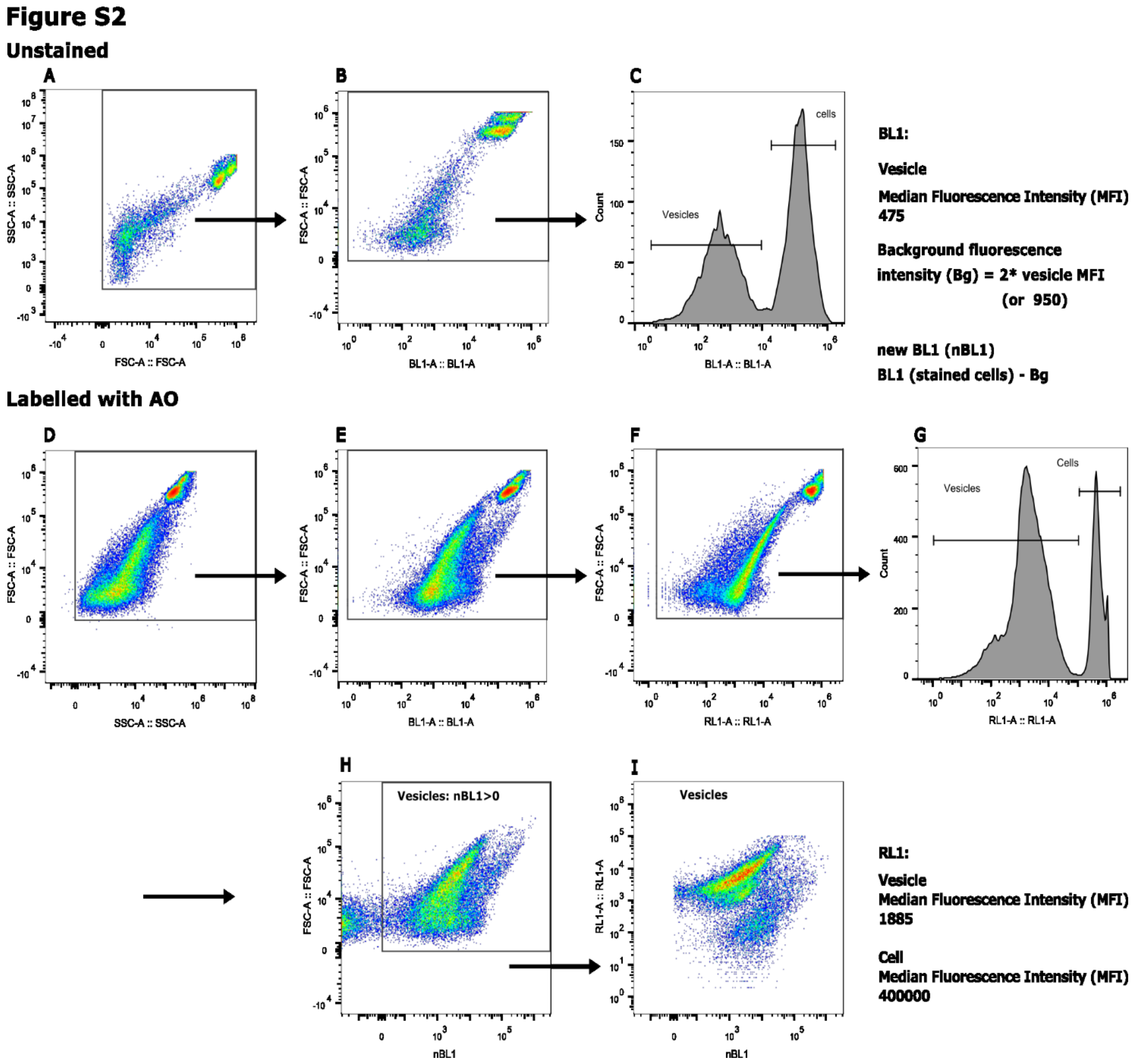

Figure S2. Vesicle identification strategy

The following strategy was followed to identify PMVs: (A-C) Events having FSC-A, SSC-A and BL1-A values greater than ‘1’ were specifically selected to measure the fluorescence of unstained vesicles. To exclude possible debris, twice the median fluorescence intensity from BL1-A of unstained vesicles was determined and considered as a background fluorescence, i.e., new BL1 (or nBL1). (D-I) Vesicles were counted only after labelling samples with AO and NMRS dyes (RL1-A). The NMRS dye (F and G) stained DNA, hence cells were significantly brighter. This dye was used to exclude cells. In the next step, background fluorescence was subtracted from BL1-A fluorescence of AO (nBL1), and the remaining events were considered as vesicles (H and I).

Figure S3

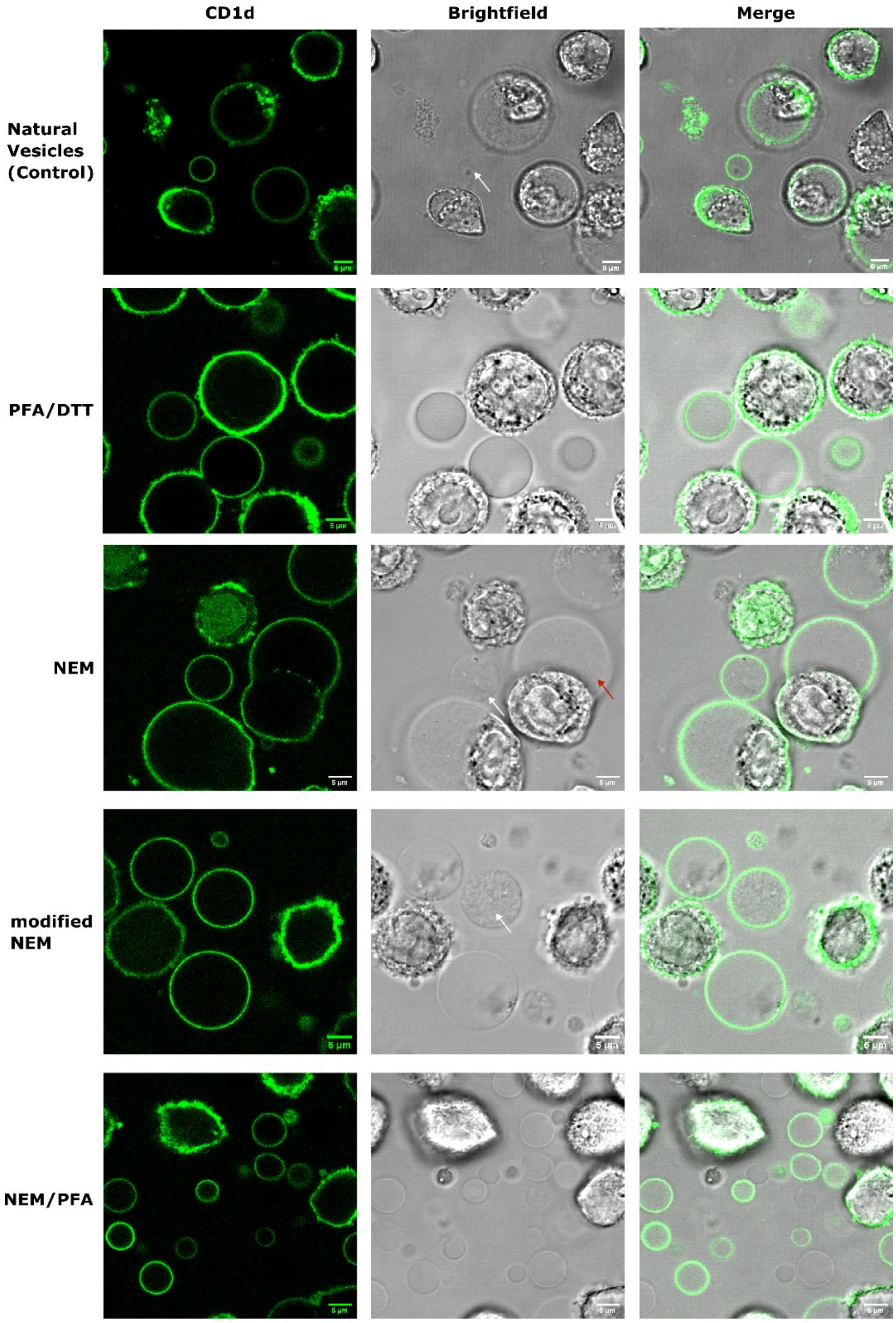

Figure S3. Fluorescent images of antibody-labelled PMVs

This figure shows confocal images of PMVs and cells stained with Alexa-488 CD1d Fabs. As expected, the staining is much brighter for cells compared to vesicles. In bright field images, cells can also be distinguished based on their intraluminal contents: PMVs are mostly transparent, whereas cells contain cellular fragments that reflect light. Vesicles show uniform circular staining for antibodies, whereas cells have non-homogeneous (or patchy) staining. Images were captured using a Zeiss LSM 880 inverted confocal microscope. Independent vesicles were observed in treatments involving PFA, whereas the NEM method resulted in a large fraction of cell-bound vesicles (third row bright field image, red arrow). Additionally, note the differences in vesicular contents, which are higher in the case of the modified NEM method (fourth row bright field image, white arrow). Scale bars are shown in the figure.
